# Analysis of trainees' memory after classroom presentations of didactical ultrasound courses

**DOI:** 10.1186/2036-7902-6-10

**Published:** 2014-07-12

**Authors:** Dorothea Hempel, Tanja Stenger, Marco Campo dell' Orto, David Stenger, Armin Seibel, Stefan Röhrig, Frank Heringer, Felix Walcher, Raoul Breitkreutz

**Affiliations:** 1 Department of Cardiology Angiology and Intensive Care, University Medical Center Mainz, Mainz, Germany; 2 Department of Internal Medicine, Municipal Hospital Neunkirchen/Saar, Neunkirchen, Germany; 3 Department of Cardiology, Kerckhoff Klinik Bad Nauheim, Bad Nauheim, Germany; 4 Regional Critical Care Ultrasound Network, Frankfurt am Main, Germany; 5 Department of Trauma, Hand, and Reconstructive Surgery, Saarland University Medical Center, Homburg/Saar, Germany; 6 Department of Anesthesiology, Intensive Care and Emergency Medicine, Diakonie Klinikum Jung Stilling, Siegen, Germany; 7 Department of Anesthesiology and Intensive Care, Marienhospital Muensterland, Krankenhaus Greven und Emsdetten, Emsdetten, Germany; 8 FINeST Frankfurt Institut of Emergency Medicine and und Simulation Training, University Hospital of the Johann Wolfgang Goethe University, Frankfurt am Main, Germany; 9 Department of Trauma, Hand, and Reconstructive surgery, University Hospital of the Johann Wolfgang Goethe University, Frankfurt am Main, Germany; 10 Department of Emergency Medicine, Klinikum Frankfurt Hoechst, Frankfurt am Main, Germany; 11 Medical Services, Lufthansa German Airlines, Frankfurt, Germany

## Abstract

**Background:**

Emergency ultrasound is gaining importance in medical education. Widespread teaching methods are frontal presentations and hands-on training. The primary goal of our study was to evaluate the impact of frontal presentations (PS) by analysis of retained knowledge rate (RKR) and learning load (LL).

**Methods:**

Our study was conducted during four introductory courses in emergency ultrasound covering Extended Focused Assessment with Sonography for Trauma (E-FAST) and Focused Echocardiography Evaluation in Life Support (FEEL). Standardized PS (length of 10 to 50 min) were presented by experienced trainers, who were asked to provide keywords, key messages, and images and assign a score to each. Group 1 consisted of 11 medical students with no prior ultrasound experience, and group 2 consisted of 80 physicians. Each group was audience to seven to eight standard PS and requested to answer a free text questionnaire after 0 h, 2.5 h, 24 h, and 14 days.

**Results:**

In group 1, 168/176 questionnaires were analyzed, and 161/202 were analyzed in group 2. RKR in group 1 was 32.5%, 15%, 16%, and 12% at 0 h, 2.5 h, 24 h, and 2 weeks. The physicians' RKR were 23%, 20.5%, and 22.4% after 0, 2.5, and 24 h of a respective PS. The LL was 1.6/min for students and 1.2/min for physicians. There was no difference in RKR when comparing PS with higher and lower LL for both groups; shorter or case-based PS were associated with a higher RKR (*p* < 0.01).

**Conclusions:**

Our study provides evidence that only a limited amount of information can be processed at a time. Only 12% of knowledge is retained after 2 weeks. Presentations of short duration can increase the retained knowledge rate. Therefore, frontal presentations and classroom-based ultrasound training and teaching should be adapted.

## Background

Ultrasound training usually consists of curricula, which include classroom-based presentations (PS) and hands-on sessions. Acquiring the theoretical knowledge and the practical skills needed to perform and interpret an ultrasound exam (knowledge, visual perception, hand-eye coordination) is a challenge for the working memory as only a limited number of items can be processed at a time [1]. The main goal of learning is to acquire knowledge and retain it over time. Apparently, most information seems to be lost within a couple of hours after the learning period as described by Ebbinghaus and others [2,3].

The time available for PS and hands-on sessions diverges between the currently available ultrasound courses. Courses offered by the German Society of Ultrasound in Medicine (DEGUM) working group for *emergency ultrasound* incorporate new learning strategies and offer online learning both pre- and post-course [4]. Blended learning not only aims to maximize the learning effect but also to optimize learning during the hands-on sessions due to the offered pre-course training. This concept with both pre- and post-course learning can enrich the ‘learning pathway’. To our knowledge, there is no literature in ultrasound education evaluating the effect of frontal PS.

This study was conducted during the standardized DEGUM course formats Extended Focused Assessment with Sonography for Trauma (E-FAST) and Focused Echo Entry Level (FEEL) offered by the emergency ultrasound section. E-FAST combines teaching of free intraabdominal fluid recognition with ultrasonographic assessment of the thorax and trachea [5-7]. FEEL courses focus on the basics of focused emergency echocardiography [8,9]. For both course formats, additional pre- and post-course learning modules are offered online by SonoABDC.org [4].

The goal of this study was to measure retained knowledge rate (RKR) after PS and analyze the influence of length or type of presentation (case-based vs. content-based).

## Methods

### Study participants

Two groups were recruited for the study. All participants were blinded to the goal of the study, participated voluntarily, and gave informed consent.

Group 1 consisted of 11 medical students who listened to seven standardized PS and filled out a questionnaire at three out of four predefined time points afterwards. The three time points were randomly chosen out of four predefined time points: immediately after the presentation (time point 1), after 2.5 h (time point 2), after 24 h (time point 3) or after 2 weeks (time point 4).

Physicians of different subspecialties who attended DEGUM emergency ultrasound courses comprised group 2 (*n* = 80). Eight PS were presented, and questionnaires had to be filled out at three predefined time points after the PS: immediately after (time point 1), after 2.5 h (time point 2), and after 24 h (time point 3). At each time point, 12 physicians were randomly chosen to answer the questionnaires.

### Presentations

All PS were prepared in a standardized format and presented by experienced trainers. The PS lasted between 10 and 50 min using a range from 12 to 42 slides. The presentations covered topics of relevance to clinical practice, and these algorithms were practiced during hands-on training, for example, ‘pleural effusions’, ‘integration of the FEEL algorithm into advanced life support’, or ‘pneumothorax’. In addition to the content-oriented PS, each group listened to two case-based presentations lasting 10 min each (six to nine slides). The cases illustrated a clinical problem/setting experienced by one of the trainers.

### Retained knowledge rate and learning load

Prior to the presentation, every trainer noted the most important information, learning contents (substantives, figures, or context) as well as key messages that he or she wanted to convey, and assigned a score from 1 to 3 regarding the importance of the information (1, important; 2, very important; 3, most important), which was then added up to a maximum score that could be achieved for a presentation. During the analysis of the participants' questionnaires, a maximum score was calculated accordingly. The score of the participant divided by the maximum score for the presentation was defined as a new variable named the ‘retained knowledge rate’.

(1) $$\begin{array}{ll} \;\mathrm{RKR}= & \mathrm{participant}\;\mathrm{score}\;\mathrm{divided}\;\mathrm{by}\;\mathrm{maximum}\\ & \mathrm{score}\;\mathrm{of}\;\mathrm{presentation}\;\left[\%\right] \end{array}$$

Furthermore, the participants were asked to reproduce the key messages. The number of learning contents of a presentation was divided by the duration of the presentation and defined as learning load (LL).

(2) $$\begin{array}{ll} \;\mathrm{LL}= & \mathrm{learning}\;\mathrm{content}\;\mathrm{divided}\;\mathrm{by}\\ & \mathrm{duration}\;\left[\mathrm{number}/\min\right] \end{array}$$

### Statistical analysis

All statistical analysis was performed using GraphPad Prism (GraphPad Software, Inc. La Jolla, CA, USA). All results are presented as quotients or mean with standard deviation. Because a normal distribution cannot be expected, differences between the groups were tested using the Mann-Whitney *U* test and the one-way RM ANOVA for more than two groups. Statistical significance was defined as *p* < 0.05.

Graphs were designed using SigmaPlot (Systat Software Inc., San Jose, CA, USA). Data are presented as box plots with 25% and 75% quartiles, upper and lower margins from 5% to 95%, and outliers, means, and medians.

## Results

Of the 11 medical students (group 1), only 2 stated that they had prior exposure to ultrasound (1 to 100 exams); the majority (n=9) had no prior knowledge about ultrasound. Of the 80 participating physicians (group 2), 77% stated that they had minimum to no experience prior to the study (47% performed 1 to 100 exams and 30% had performed no ultrasound exams at all).

In group 1, 168 out of 176 questionnaires were available for analysis (95.9%). Eight questionnaires had to be excluded due to missing information. In group 2, 161 of 202 (79.7%) questionnaires were analyzed.

**Figure 1:**
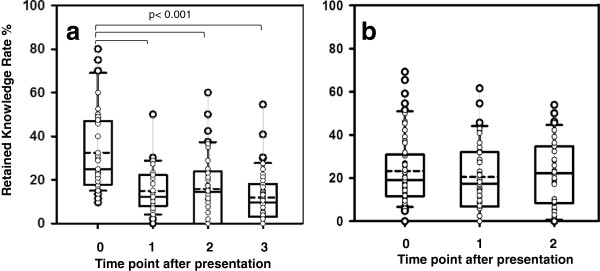
**RKR in groups 1 (a) and 2 (b) at different time points of analysis after the presentation. (a)** Group 1: RKR was markedly reduced in medical students after frontal presentations at time points 0 to 3 (0 h, 2.5 h, 24 h, and 14 days). **(b)** Group 2: RKR, in percent, at time points 0 to 2 (0, 2.5, and 24 h). There was no difference between the time points.

**Figure 2:**
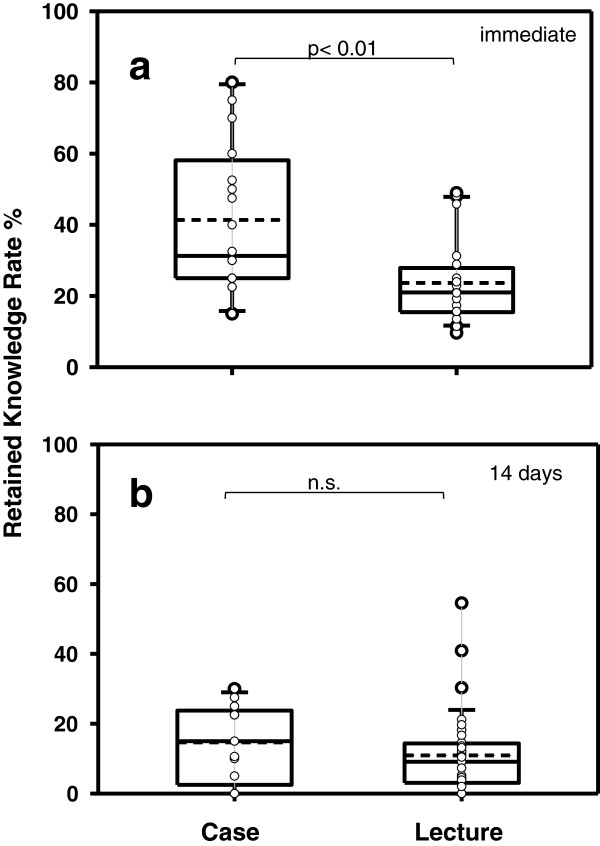
**Comparison of RKR between case-based and standardized didactical lectures in group 1. (a)** Immediately after presentations (time point 0) and **(b)** 14 days later.

The RKR at time point 0 was 33% for group 1 and 23% for group 2, declining to 12% after 14 days in group 1 (Figure 1a,b). After case-based presentations, the RKR at time point 0 was significantly higher when compared to standard PS in groups 1 (Figure 2a) and 2. In group 1, no difference was seen at all later time points (Figure 2b), whereas in group 2, a significant difference was measured after 2.5 h, but not after 24 h (Table 1).

**Table 1:** Comparison of RKR for case-based and standard PS for group 2 after 0, 2.5, and 24 h

**Time point after PS (h)**	**RKR (%)**		***p *** **value**
	**Case-based PS**	**Standard PS**	
0	32.8 ± 4	19.9 ± 2	<0.01
2.5	26 ± 3.5	17 ± 3.4	<0.01
24	21.5 ± 4	22.8 ± 3.1	n.s.

n.s., not significant.

**Figure 3:**
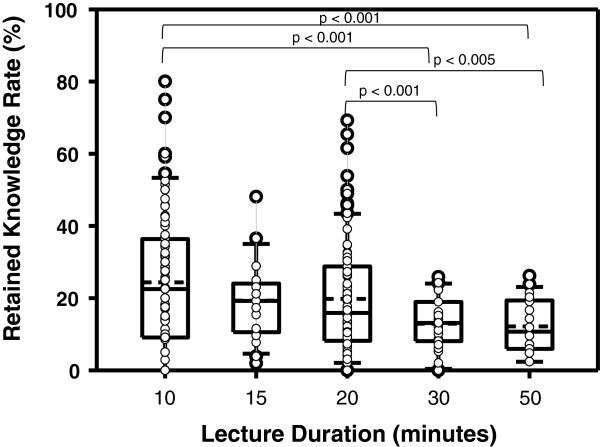
Pooled data of both groups for correlation of retained knowledge rate with various lengths of presentations.

The average LL was 1.6 learning contents/min in group 1 and 1.2 learning contents/min in group 2. When comparing the RKR of PS with lower and higher LL, no difference was found: In group 1, the RKR (in percent) after presentations with a LL < 1.1 was 22.3 ± 3.6 compared to 17.3 ± 1.2 for PS with a LL > 1.1 (not significant). In group 2, the RKR after PS with a LL < 1.2 was 24.1 ± 1.7 compared to 18.4 ± 1.6 for PPs with a LL > 1.2 (not significant). In both groups, shorter PS showed a higher RKR when compared to longer presentations (Figure 3).

Immediately after the PS, analysis of retention of key messages showed a significantly higher RKR in both groups when compared to other learning content provided in the PS. At later time points, no differences were found regarding group 1. In group 2, the RKR was higher when compared to other learning contents at all time points (Table 2).

**Table 2:** Comparison of RKR for key messages and learning content at various time points in groups 1 and 2

**Time point**	**Group 1**			**Group 2**		
	**RKR key**	**RKR content**	***p *** **value**	**RKR key**	**RKR content**	***p *** **value**
0 h	43.6 ± 4,4	32.5 ± 3.1	<0.05	41.9 ± 2.9	23.3 ± 1.9	<0.05
2.5 h	19.5 ± 3,2	15.0 ± 1.6	n.s.	32.7 ± 3.9	20.5 ± 2.5	<0.05
24 h	22.0 ± 3,7	16.0 ± 2.4	n.s.	43.5 ± 4.5	22.4 ± 2.4	<0.05
14 days	20.3 ± 3,4	12.0 ± 1.7	n.s.			

The RKR for key messages was higher in group 2 when compared to learning content at all time points. RKR key, retained knowledge rate for key messages; RKR content, retained knowledge rate for learning content; n.s., not significant.

## Discussion

In this study, we aimed to evaluate the influence of the length of a PS, the learning load, and the format by analyzing the retained knowledge rate after standardized presentations. Our main findings are that in longer-term memory up to 14 days, the retained knowledge rate of PS is very weak, corresponding to less than 20% of the content that was originally presented and classified as a learning goal by a trainer. Furthermore, case-based presentations showed no improvement in retaining information. Surprisingly, learning load had no influence on the rate of retained knowledge, and there was no inverse correlation with respect to the length of the presentation.

Current ultrasound teaching almost always incorporates lectures and hands-on training. Presentations are widely used in ultrasound classroom teaching, but there are many critics pointing out that many connections are not displayed correctly due to widely used abbreviations and bulleted lists on the slides [10]. The phenomenon that presentations seem tedious and dull due to an overload of information and bad layout often has been called ‘death by PowerPoint’ [11,12]. There is contradicting evidence about the use of multimedia presentations in education. Some studies comparing multimedia to overhead slides showed no difference in students' test results [13,14]. Other studies were able to show that students performed better in multiple-choice test if they had listened to lectures using multimedia slides [15]. Lowry et al. were able to show that the amount of information presented on the slides had no influence on students' test performances [16].

To our knowledge, we present the first study analyzing the use of standardized PS in teaching ultrasound. All PS used during this study were developed according to the cognitive load theory of Sweller [17,18]. This theory implies that there are two channels processing information: the visual and the auditory channel. Both lead the information into the working memory, which only has a limited capacity (cognitive load) to process information. According to Sweller's theory, cognitive load can be divided into three forms: (1) The extraneous load, which represents the unnecessary information not absolutely needed, such as visual animations for better visualization, should be reduced to a minimum so as not to burden the working memory. (2) The intrinsic load represents information that consists of the complexity of the learning content such as diagrams and flowcharts. (3) The germane load denominates the capacity that the trainee needs to process the information into new schemes. The germane load can be reduced by a conclusive presentation. The most important information was denominated as such by the lecturers and repeated at the end of the presentation. Although all the aforementioned aspects were incorporated in the PS, the retained knowledge rate in our study was low.

We analyzed the effect of the teaching intervention by measuring the RKR that was defined as the score of the participant divided by the possible maximum score of the presentation as defined by the lecturer (%). The RKR immediately after the presentations in both groups was low and showed the typical decline as presented by the forgetting curve of Ebbinghaus [3] for the group of students. The initial RKR of the postgraduate group was lower, which could be explained by a lower motivation to participate in the study. Another explanation is that students are trained to listen to and process information presented in the form of multimedia frontal presentations on a daily basis. As the study was performed during the introductory period of emergency ultrasound courses in Germany and a fee was charged for participation, we may assume that all participants were highly motivated. In a different study, participants were asked about their pre-course learning strategies. Participants stated that they would start preparation about 4 weeks prior to the course. Both may be interpreted as a high motivation.

The effect of case-based presentations has not been studied in ultrasound education so far. In our study, the use of case-based PS showed an increase of RKR in the short-term but obviously not reaching the long-term memory. We speculate that incorporation of cases into hands-on sessions might have a more profound effect.

In our study, there was an inverse relation between the length of a presentation and the RKR, meaning that the shorter the PS, the higher the RKR. This corresponds well to a study of Hofer et al. concluding that medical students preferred short PS and that a duration of 20 min should not be exceeded for ultrasound lectures.

We defined the learning load as learning content divided by duration (number/min). In our study we, unexpectedly could not find a significant difference between presentations with higher and lower LL. That corresponds to a study of Lowry et al. that showed no correlation of the amount of information on a slide and the students' achievement in a multiple-choice test [15]. However, further study is needed to analyze if an even higher LL of more than two items per minute remains without influence on the RKR.

Key messages were defined as content that was most important to the trainer. When comparing the RKR for overall learning content and key messages, the RKR for key messages was significantly higher at all time points in the postgraduate group. In the student group, the slope according to the forgetting curve of Ebbinghaus was seen again. The difference could be explained by the lack of clinical experience in the student group making it more difficult to recognize key messages. The students would then more likely be prone to direct their attention at to more detailed information not crucial to the general concept. Therefore, a PS can be improved by highlighting and repeating key messages at the end of every presentation.

With respect to the results of our study, our suggestion for future ultrasound courses would be to incorporate shorter presentations of less than 20 min with up to five key messages. Theoretical and hands-on sessions should either alternate or be divided into a theoretical morning session and a hands-on session in the afternoon. Alternating between theory and hands-on session might prevent fatigue and enhance retention of knowledge [19]. However, an alternating program has a higher staff expense as all trainers are required to be available all day and be able to teach every topic. Pre- and post-course training should be incorporated, for example, in the form of webcasts to define a learning pathway and repeat important learning targets using a blended learning concept.

Special courses for lecturers in ultrasound education have recently been established [20]. We believe that special courses for ultrasound trainers in addition to short sessions during ultrasound courses have the potential to better promote learning.

### Limitations

Our study is limited as only theoretical knowledge was tested and no evaluation of practical skills was performed. Due to the number of participants of the courses, the number of study participants is not equal between the student and the postgraduate groups. We did not assess motivation of the participants before the study. Although all teachers were highly trained and experienced and presentations were designed in a standardized fashion, we did not assess the influence the individual teacher had on the learning effect.

## Conclusions

Our study provides evidence that a limited amount of information can be processed at a time in classroom teaching of ultrasound courses. Only 12% of knowledge is retained after 2 weeks. Presentations of short duration and case-based presentations can increase the retained knowledge rate. Therefore, frontal presentations and classroom-based ultrasound training and teaching should be adapted in the future.

## Competing interests

The ultrasound regional network SonoABCD.org supported this study. The authors declare that they have no competing interests.

## Authors' contributions

DH and TS contributed equally and share first authorship. All authors contributed significantly to the manuscript and read and approved the final manuscript.
